# Glomus Cell Tumors: Demographic and Clinical Characteristics

**DOI:** 10.7759/cureus.84615

**Published:** 2025-05-22

**Authors:** Carla Itzel Figueroa-Basurto, Daniela Attili Castro, Miren Lorea Cárdenas Hernández, Elisa Vega-Memije, Ana L Ramirez Teran

**Affiliations:** 1 Dermatology, Hospital General "Dr. Manuel Gea González", Mexico City, MEX; 2 Dermatology, Skin Institute, Mexico City, MEX; 3 Dermatopathology, Hospital General "Dr. Manuel Gea González", Mexico City, MEX

**Keywords:** demographic data, glomangioma, glomus tumors, hospital epidemiology, rare clinical presentation

## Abstract

Introduction: Glomus tumors and glomangiomas are neoplasms derived from modified smooth muscle cells, primarily located in acral skin. They are benign and rare. Glomus tumors typically appear between the ages of 20-40, with 90% being solitary. They are usually accompanied by pulsating pain. Glomangiomas are more common in females, are located on the trunk and extremities, usually asymptomatic.

Objective: Describe the demographic and clinical characteristics of glomus tumors and glomangiomas in a general hospital of tertiary care, based on cases studied from January 1994 to December 2023.

Methodology: An observational, descriptive, retrospective, and cross-sectional study was conducted using the electronic database of the Dermatopathology Department of General Hospital "Dr. Manuel Gea González" from January 1994 to December 2023.

Results: The study population comprised 63 patients with 69 glomus cell tumors. Among these, 36 lesions were identified in 36 patients - 26 women and 10 men - with an average age of 50.19 years. The most commonly affected anatomical site was the nail bed, with pain being the predominant symptom. Regarding glomangiomas, 33 lesions were recorded in 28 patients - 7 women and 21 men - with an average age of 43.4 years. The forearm was the most frequently affected site, and pain remained the predominant symptom.

Conclusions: Glomus tumors and glomangiomas are painful tumors, with glomus tumors favoring the nail bed and glomangiomas the extremities. Despite their morphological similarities, they should be considered in the differential diagnosis of painful tumors. Accurate histological diagnosis is essential to properly characterize these tumors and avoid confusion. Providing epidemiological and clinical information about these tumors is important.

## Introduction

Glomus tumors and glomangiomas are neoplasms derived from modified smooth muscle cells found in glomus bodies, or the Sucquet-Hoyer canal, primarily located in acral skin. They are generally benign and uncommon.

The 2020 World Health Organization (WHO) classification of soft tissue tumors groups glomus cell tumors under perivascular (pericytic) tumors, including glomus tumor, glomangiomatosis, and malignant glomus tumor.

Glomus tumors typically appear between the ages of 20 and 40, with 90% being solitary and 75% located in the hand, of which 65% are subungual [4,5]. Most are accompanied by pulsating pain radiating to the extremity, exacerbated by pressure and temperature changes, especially at night [4,6,7]. Histologically, they present as well-defined, non-encapsulated, solid dermal neoplasms composed of round and cuboidal glomus cells with pale eosinophilic cytoplasm surrounding thin-walled capillaries [8]. Dermatoscopy aids in tumor delineation and visualization of the vascular pattern suggestive of a glomus tumor [9].

Glomangiomas, or glomus venous malformations, are benign vascular skin lesions characterized by irregular venous channels surrounded by glomus cells [10-13]. They are more common in women, during childhood, and between the fourth and fifth decades of life, primarily located on the trunk and extremities, and are asymptomatic [11-14]. They are classified into three types: multiple disseminated, multiple localized, and congenital plaque glomangioma, the rarest type [15].

Both tumors are treated surgically, with a 20% recurrence rate [16].

The objective was to describe the demographic and clinical characteristics of glomus tumors and glomangiomas in a general hospital of tertiary care, based on cases studied from January 1994 to December 2023.

## Materials and methods

An observational, descriptive, retrospective, and cross-sectional study was conducted using the electronic database of the Dermatopathology Department at the General Hospital "Dr. Manuel Gea González", located in Mexico City, Mexico. Cases diagnosed histologically as glomangioma, glomus tumor, or glomus cell tumor between January 1994 and December 2023 were reviewed. A non-probability convenience sampling technique was employed, based on the availability of cases within the institutional database. Inclusion criteria were as follows: confirmed histopathological diagnosis of glomangioma, glomus tumor, or glomus cell tumor; availability of complete demographic and clinical data, including time of evolution, lesion topography, morphology, and symptoms; and patients of any age or sex. Exclusion criteria comprised inconclusive or questionable histopathological diagnoses, incomplete clinical records, reclassified lesions, and duplicate entries. Descriptive statistical analysis was performed using Microsoft Excel 2019 ®, calculating mean, median, and mode for continuous variables, and frequencies and percentages for categorical variables. An observational, descriptive, retrospective, and cross-sectional study was conducted. The electronic database of the Dermatopathology Department of the General Hospital "Dr. Manuel Gea González" was used. Cases with a histological diagnosis of glomangioma, glomus tumor, and glomus cell tumor were selected. We included those that had demographic information, time of evolution, topography, morphology, and symptoms between January 1994 and December 2023.

## Results

The total population was 63 patients with 69 glomus cell tumors. One patient had both tumors in different topographies and at different times. Table 1 shows a summary of the main findings of our study.

**Table 1:** Summary of main findings. This table presents a summary of the main results, including a total of 69 lesions identified in 63 patients.

Variables	Glomus tumor	Glomangioma
Patients, *n *(%)	36 (57%)	28 (43%)
Lesions, *n* (%)	36 (52.17%)	33 (47.82%)
Mean age (years)	50.19	43.4
Sex most frequently affected (%)	Women (72.22%)	Men (75%)
Most common location (%)	Nail bed of the index finger (61.11%)	Right forearm (18.18%)
Pain, *n* (%)	33 (94%)	25 (78.7%)

Glomus tumor 

Glomus tumors were identified in 36 patients (52.17%), comprising 26 women (72.22%) and 10 men (27.78%), with a mean age of 50.19 years (range: 23-82 years).

**Figure 1:**
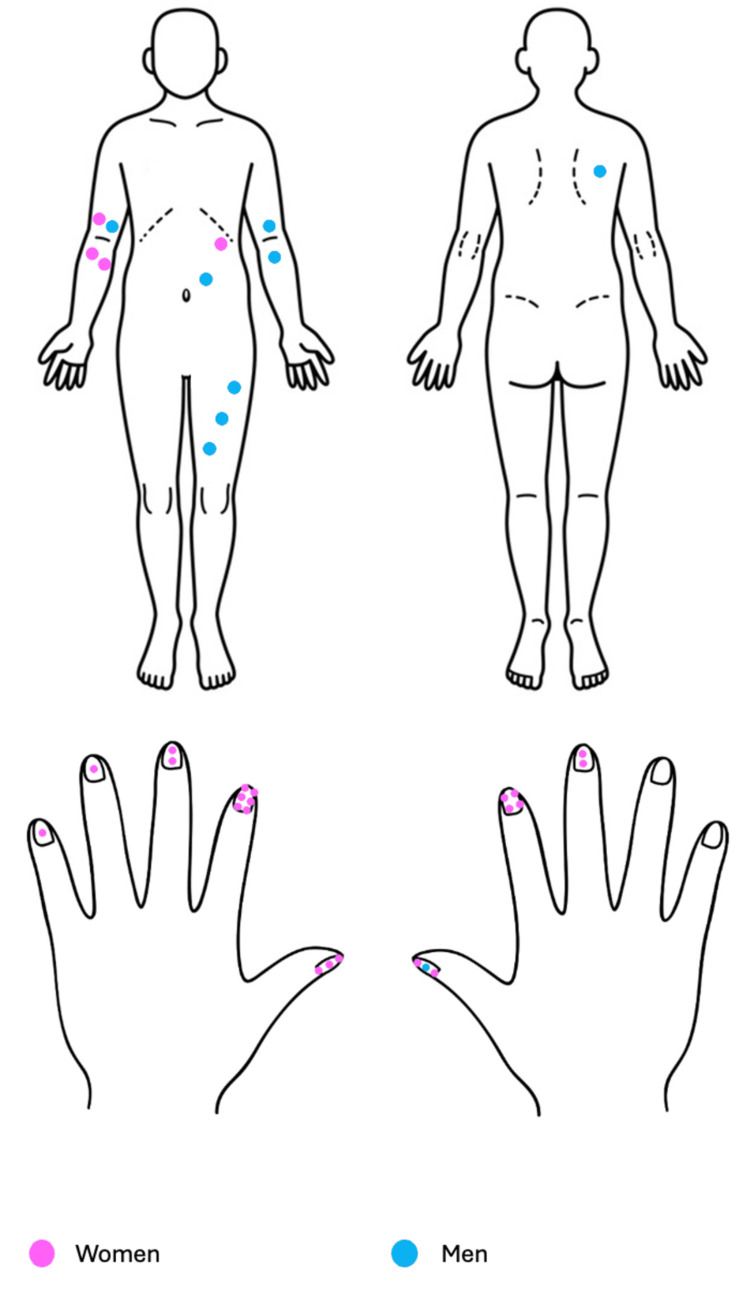
Distribution of the 36 cases of glomus tumor. Thirty-six lesions (52.17%) were identified in 36 patients: 26 women (72.22%) and 10 men (27.78%). Lesions were located on the trunk (3), upper extremities (29), and lower extremities (4); in two cases laterality was not specified. The most affected site was the nail bed of the index finger (10 lesions). Image adapted by the authors from: https://www.dreamstime.com/stock-illustration-front-back-human-body-image71473837

Lesions were located as follows: trunk (3), upper extremities (29), and lower extremities (4). Seventeen lesions were on each side; laterality was not specified in two cases. The most commonly affected site was the nail bed of the index finger (10) (Figure 1).

Tumors in the nail apparatus were described as bands of erythronychia and/or bulging of the nail plate; elsewhere, they appeared as subcutaneous, exophytic neoformations without epidermal changes. The mean size was 0.5 cm, and the duration of evolution was 4.9 years (range: 0.08-15 years). Thirty‑three patients (94%) reported pain.

**Figure 2:**
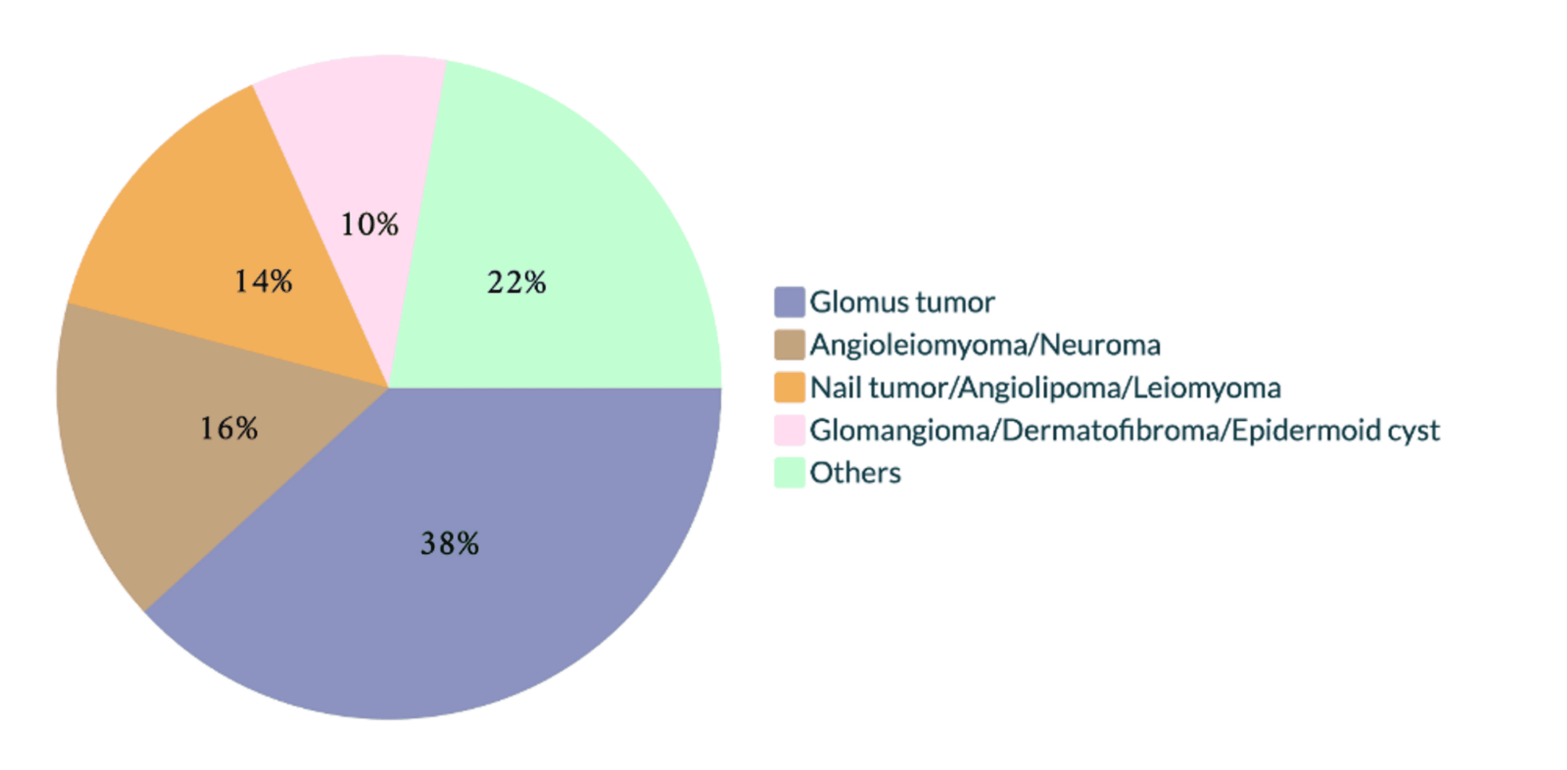
Differential clinical diagnoses submitted for glomus tumor. The other diagnoses issued on one occasion were fibrokeratoma, pyogenic granuloma, foreign body granuloma, atypical mycobacteriosis, spiradenoma, onycholysis, neurofibroma, hemangioma, angioma, Masson's tumor, lipoma, mucoid cyst, common wart, and squamous cell carcinoma.

Glomus tumor was the primary diagnosis in 17 of 36 cases (47.22%), and the sole diagnosis in 14 of these (82.35% diagnostic certainty). The remaining differential diagnoses are shown in Figure 2.

In all cases, the approach was surgical. Four patients experienced recurrence, with a reappearance time of 4 (0.08-8) years. Two recurrences were in the nail bed, one in the abdomen, and the other on the back. The latter was reported as an atypical glomus tumor with focal necrosis, and the recurrence was identified as a malignant glomus tumor.

Glomangioma

**Figure 3:**
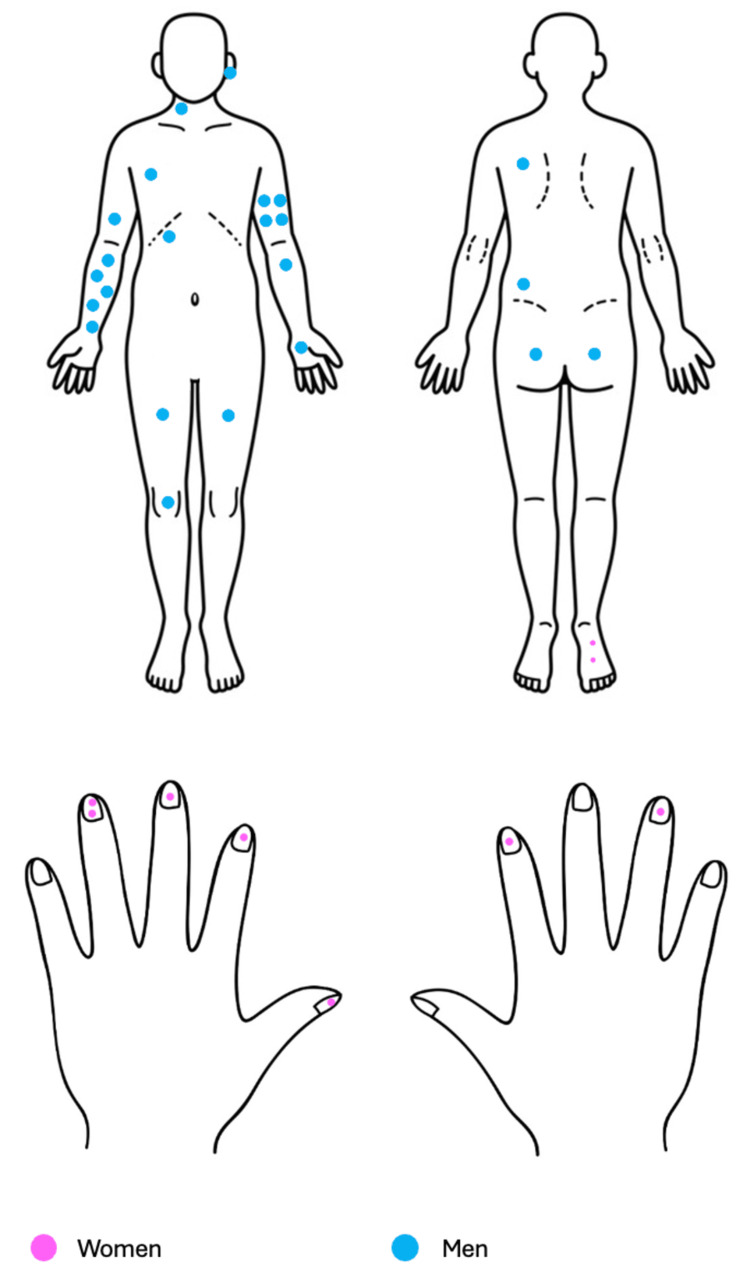
Distribution of the 33 cases of glomangioma. Thirty-three glomangiomas (47.82%) were recorded in 28 patients (43%): 7 women (25%) and 21 men (75%). Lesions were located on the head and neck (2), trunk (4), upper extremities (20), and lower extremities (7); laterality: right side (14), left side (18), and unspecified (1). The most affected site was the right forearm. Image adapted by the authors from: https://www.imagui.com/a/imagenes-para-dibujar-de-manos-TKdAkj4zy#google_vignette

There were 33 (47.82%) glomangiomas recorded in 28 patients: 7 (25%) women and 21 (75%) men, with an average age of 43.4 years (range: 23-75). Two patients had multiple glomangiomas: one with two lesions and the other with five, both diagnosed simultaneously. Lesion distribution was as follows: head and neck (2), trunk (4), upper extremities (20), and lower extremities (7); laterality: right side (14), left side (18), and unspecified (1). The most affected site was the right forearm (5 lesions) (Figure 3).

The tumors were described as exophytic, dome‑shaped, poorly defined neoplasms with an erythematous‑violaceous color and a mean size of 0.45 cm. The duration of evolution was 5.7 years (range: 0.25-20 years), and 78.7% of patients reported pain.

**Figure 4:**
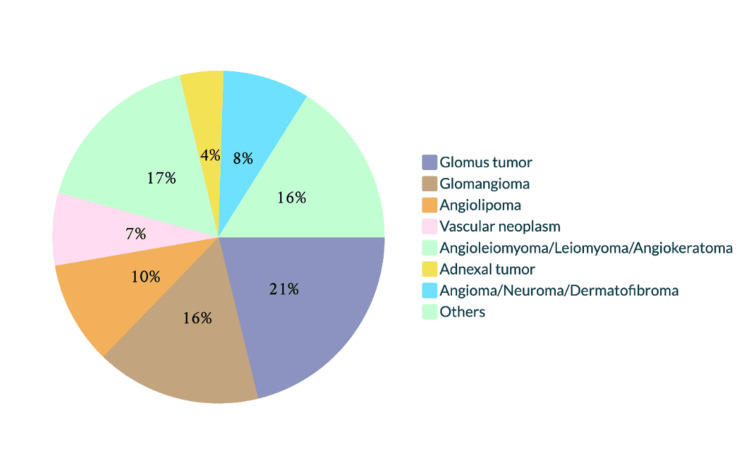
Differential clinical diagnoses submitted for glomangioma. The other diagnoses, each made on a single occasion, were neurofibroma, vascular tumor, venous aneurysm, pseudosarcoma, Kaposi sarcoma, hemangioma, spiradenoma, angiosarcoma, blue nevus, and pyogenic granuloma.

In 7 of 33 cases (21.2%), glomangioma was the primary diagnosis, and in 2 of those 7 cases (28.6%), it was the sole diagnosis. The other differential diagnoses are shown in Figure 4.

All cases were treated surgically. One patient experienced recurrence one year later.

## Discussion

The glomus body is a contractile neuromyarterial receptor composed of glomus cells that regulate flow, pressure, and temperature in the cutaneous microvasculature and can give rise to glomus tumors and glomangiomas [17]. When these tumors present outside the skin, it is considered that they originate from ectopic glomus cells or undifferentiated perivascular cells with the capacity to transform into glomus cells [1].

In this study, we identified 69 glomus cell tumors over a period during which 42,761 histopathological reports were issued. Of these, 36 were glomus tumors (0.08%) and 33 were glomangiomas (0.07%). This translates to 1.25 glomus tumors and 1.14 glomangiomas per year. Karam-Orantes et al. reported a 0.17% incidence of glomus tumors in a series of 9,436 biopsies over six years [5]. In the thesis by Pérez, 1.65 glomus tumors and 0.05 glomangiomas were reported over 64 years [18]. Galicia-Maldonado et al. reported an annual incidence of 0.6 subungual glomus tumors in a 10-year series [19]. These data suggest that these neoplasms are relatively rare; however, they are significant due to the symptoms they produce and their locations, which can affect the patient's quality of life. 

The female-to-male ratio for glomus tumors in our study was 2.6:1, consistent with the literature. The average age in our series was 50 years, a decade older than reported in other studies [20-24]. Regarding glomangiomas, our series predominantly featured male patients (3:1), which differs from the literature. The age at diagnosis was 43 years, similar to other reports [10,11,13,18,25,26].

Glomus tumors can appear anywhere on the body, with the most common site being subungual [1,2,4,6,7,23,24,25]. In our study, almost 70% of glomus tumors were subungual. Glomangiomas predominantly affect the extremities and trunk [11-14]. In our series, almost 94% were in these areas.

Morphologically, both tumors have similar characteristics in tenderness, size, and shape. The main reported difference is in color: glomus tumors have bands of erythronychia, while glomangiomas are erythematous-violaceous [2,4,10,11,18], consistent with our findings.

In our cases, 94% of glomus tumors and 78% of glomangiomas were painful, whereas the literature often describes glomangiomas as asymptomatic [10,11,18].

The reported evolution time in the literature for glomus tumors is 3.3 years (range: 0.3-10 years) [4,6,7], similar to our findings. For glomangiomas, the reported evolution time is between 10 and 13 years, as they are usually asymptomatic [10-13,25,27], which contrasts with our series.

Mravic et al. reported a clinicopathological concordance of 45.4% for glomus tumors [20]. In our study, the certainty was 28.57% for glomangiomas and 82% for glomus tumors. In their series, the main differential diagnoses were lipoma and cyst, whereas in our study, the main differential diagnoses were angioleiomyoma and neuroma, both of which are included among painful tumors.

As a retrospective review of cases collected over nearly three decades, there is a potential for selection bias, since only patients who were biopsied and recorded in the dermatopathology database were included. Additionally, information bias may be present due to inconsistencies or omissions in historical clinical records. The study being conducted in a single tertiary care center may also introduce referral bias, as more complex or atypical cases are more likely to be referred, potentially affecting the generalizability of the findings. Finally, the sample size, while notable for the rarity of glomus tumors, remains relatively small for robust statistical inference.

Our findings suggest that clinicians should maintain a high index of suspicion for glomus tumors and glomangiomas, particularly in patients presenting with chronic localized pain, subungual lesions, or vascular-appearing nodules on the extremities and trunk. Given the frequent misdiagnosis and long evolution time observed, especially in glomangiomas, early dermatological evaluation and biopsy are crucial to ensure accurate diagnosis and effective treatment. The high clinicopathological concordance for glomus tumors also reinforces the importance of clinical-pathological correlation in improving diagnostic precision. Furthermore, awareness of the demographic and anatomical patterns observed in our series may aid clinicians in recognizing these tumors more promptly, ultimately improving patient outcomes and quality of life.

## Conclusions

Glomus tumors and glomangiomas are painful tumors, with the former predominantly affecting the nail bed and the extremities, respectively. Morphologically, they share similarities and should be considered in the differential diagnosis of painful tumors alongside other entities like eccrine spiradenoma, neurilemoma, glomus tumor, leiomyoma, angiolipoma, neuroma, and dermatofibroma (ENGLAND). 

Most of these tumors are benign and can be effectively treated with complete excision; however, there is a risk of recurrence and malignant transformation. Histology plays a crucial role in accurately diagnosing both entities, allowing for proper characterization and avoiding diagnostic confusion. We believe it is important to contribute epidemiological and clinical information about these fascinating tumors.
